# Epilepsy lifetime prevalence in Iran: a large population- based national survey

**DOI:** 10.1038/s41598-021-89048-z

**Published:** 2021-05-03

**Authors:** Hossein Pakdaman, Ali Amini Harandi, Koroush Gharagozli, Farshid Alaeddini, Akram Esfandani, Seyed Hamidreza Mirbehbahani, Taher Doroudi, Pirhossein Kolivand, Parviz Bahrami, Hadi Kazemi

**Affiliations:** 1 Brain Mapping Research Center, Shahid Beheshti University of Medical Sciences, Tehran, Iran; 2 Shefa Neuroscience Research Center, Khatam-Ol-Anbia Hospital, Tehran, Iran

**Keywords:** Medical research, Neurology

## Abstract

Epilepsy has garnered increased public health focus because patients who suffer from epilepsy experience pronounced and persistent health and socioeconomic disparities despite treatment and care advances. The epidemiology of epilepsy is diverse in different countries and regions. This nationwide population-based cross-sectional study was conducted to determine the life time prevalence and health related factors of epilepsy for the first time in Iran through a two-phase door-to-door survey method. In phase I, a screening for epilepsy was performed on 68,035 people. Then in phase II, after the neurological evaluation of participants and reviewing medical records, 1130 subjects with epilepsy was confirmed. The life time prevalence of epilepsy was achieved to be 16.6 per 1000 people (95% CI 15.4–17.8) with the average age onset 19.1 ± 21.1 (active prevalence 9.5 per 1000 people). Focal seizure (59.3%), generalized epilepsy (38%) and unknown types of epilepsy (2.7%) were detected among participants. The overall life time prevalence of febrile convulsion was 4.1 per 1000 people. The frequency of attacks per year and per month were 3.0 ± 1.6 and 0.5 ± 0.1, respectively. Age-specific life time prevalence was highest among the age group of 15–19 years old [32.7 per 1000 persons (95% CI 29.1–36.8)] and it was higher in male (53.8%) than female (46.2%) participants. Our results showed that the life time prevalence of epilepsy in Iran is higher than worldwide average.

## Introduction

Epilepsy has garnered increased public health focus because patients who suffer from epilepsy experience pronounced and persistent health and socioeconomic disparities despite treatment and care advances. The epidemiology of epilepsy is diverse in different countries and regions^1^. The lifetime prevalence of epilepsy varies between 3.5 and 10.7 per 1000 persons in developed countries, and from 0.9 to 74.4 per 1000 persons in Asia, sub-Saharan Africa, and Latin America^2,3^. Furthermore, epilepsy life time prevalence is higher in rural areas than urban centers^4^. Vast numbers of risk factors besides miscellaneous methodology are partially at fault of above-mentioned differences in epidemiology of epilepsy in previous researches. In addition, diagnosis of epilepsy is highly dependent on the patient history and in the lack of a precise route, differences in the criteria that were utilized in the surveys intensify the differences in the epidemiological findings.

The epidemiological and clinical features of the disorder are diverse in different races and ethnicities. Although there are various reports on prevalence of epilepsy in different regions, large nationwide survey in the epidemiology of epilepsy has not conducted in Iran. Iran is one of the most influential middle-east-located country in its region with the income level 3 since 1955, so far and little has been discovered about the epidemiological and clinical features of epilepsy in Iranian population. In the light of previous sparse-population studies that were proposed in Iran, life time prevalence of epilepsy was estimated to be circa 50 per 1000 people in a meta-analysis^5^.

The present study is the first nationwide study which provided updated national and modeled state-specific numbers of active epilepsy cases. Moreover, this survey was proposed in order to clarify and determine the life time prevalence of epilepsy among both sexes, besides the most common risk factors, etiologies, the mean age of onset of epilepsy, the pharmacotherapy approach of Iranian neurologists and the average expenditure paid off by the patients.

Public health practitioners, health care providers, policy makers, epilepsy researchers, and other epilepsy stakeholders, including family members and people with epilepsy, can use these findings to ensure that evidence-based programs meet the complex needs of adults and children with epilepsy and reduce the disparities resulting from it.

## Results

**Figure 1:**
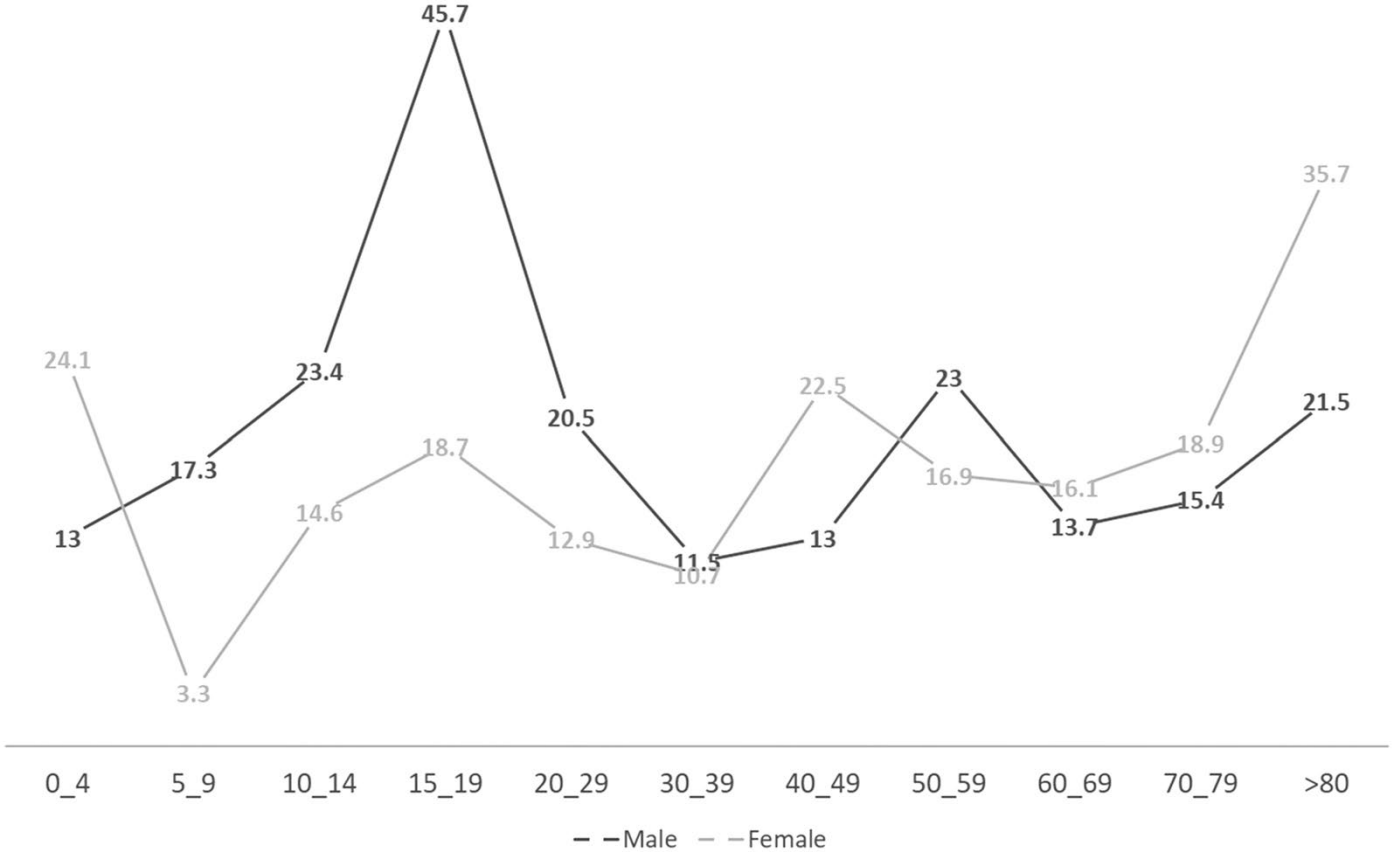
The life time prevalence of epilepsy in age and sex groups.

Finally, 68,035 residents of considered areas of the present study were screened in the first phase of the study (Table 1). The mean age of participants was 36.2 ± 19.8 (mean ± SD). The mean years of education was 8.4 ± 7.6 and 50.4% of participants were male. Moreover, 80.2% of persons were from urban areas. Overall, 5.8% of the individuals were positive responders (who answered ‘yes’ to at least one of the epilepsy-related questions of the questionnaire). Our analysis revealed a remarkable correlation between the age and sex of the positive responders as compared with the age (*p* < 0.0001) and sex (*p* = 0.038) of the negative responders (who answered ‘no’ to all of the epilepsy-related questions) (Table 2 and Fig. 1).

**Table 1:** Demographics of the participants.

**Gender N (%)**	
Male (34,290)	50.4%
Female (33,745)	49.6%
**Age (year)**	
Mean (SD)	36.2 ± 19.8
Urban residents (54,564)	80.2%
Rural residents (13,471)	19.8%
Mean years of education (SD)	8.4 ± 7.6

**Table 2:** Correlations between sex and age of participants on the life time prevalence.

Age groups (year)	Male (per 1000 persons)	Female (per 1000 persons)	Total (per 1000 persons)
0–4	13.0	24.1	18.8
5–9	17.3	3.3	10.8
10–14	23.4	14.6	19.2
15–19	45.7	18.7	32.7
20–29	20.5	12.9	16.6
30–39	11.5	10.7	11.1
40–49	13.0	22.5	17.8
50–59	23.0	16.9	19.8
60–69	13.7	16.1	14.9
70–79	15.4	18.9	17.0
≥ 80	21.5	35.7	27.1
Life-time prevalence	17.8 (16.0–19.5 CI 95%)	15.5 (13.8–17.2 CI 95%)	16.6 (15.4–17.8 CI 95%)

At second phase positive screened subjects were reviewed by expert neurologists and 1130 patients was confirmed as epileptic according to inclusion criteria in which 608 (53.8%) were male and 522 (46.2%) were female. Accordingly, the life time prevalence of epilepsy was 16.6 per 1000 people (95% CI 15.4–17.8) in Iran and active prevalence was 57.4% (9.5 per 1000 people). In male participants the life time prevalence was 17.8 per 1000 people (95% CI 16.0–19.5). It was calculated 15.5 per 1000 persons (95% CI 13.8–17.2) in female group. There was no difference between male and female in prevalence. The average age of epilepsy onset was 19.1 ± 21.1. The frequency of attack per year and per month were 3.0 ± 1.6 and 0.5 ± 0.1, respectively.

About 67% of patients were symptom free since the last year in which 23% of them was in remission for at least two years. It was revealed that 18.8% of patients suffered from refractory epilepsy (i.e., more than 2 seizures per year). In the last year, 14.2% of patients had one attack. Moreover, we found that 5.8% patients had more than 1 attacks per month.

In addition to that, our data demonstrated that most of the patients (59.3%) suffered from focal epilepsy, in which 42.3% of them complicated to generalized seizure. About 28.3% and 21.7% of patients who suffered from partial seizure, declared that their attacks were manifested by staring and aura, respectively. Generalized epilepsy was found in 38% patients. In approximately 2.7% of patients were of unknown types of epilepsy. The overall life time prevalence of febrile convulsion was 4.1 per 1000 people (4.8 per 1000 in male and 3.4 per 1000 in female patients) (Table 3).

**Table 3:** Types of epilepsy.

Types of epilepsy	Total N(%; CI 95%)	Male (CI 95%)	Female (CI 95%)
Generalized epilepsy	429 (38%; 36.0–40.0)	37.9% (35.3–40.7)	38% (35.0–41.0)
Focal epilepsy	670 (59.3%; 57.3–61.3)	59% (56.2–61.8)	59.6% (56.6–62.6)
Unknown	31 (2.7%; 2.0–3.4)	3.1% (2.2–4.0)	2.4% (1.4–3.4)

Age-specific life time prevalence was highest among the age group of 15–19 years old [32.7 per 1000 persons (95% CI 29.1–36.8)] and it was remarkably higher in male participants [45.7 per 1000 persons (95% CI 39.6–50.0)] than female participants (18.7 per 1000 persons (95% CI 17.0–19.7)). In view of aetiology, we determined an underlying aetiology in 50.6% of patients which could be categorised in of the following aetiological groups including trauma (21.9%), stroke (7.6%), infectious diseases (5.7%), brain tumours (5.3%) and others (10.1%). Therefore, trauma including head trauma and neurosurgical complications was determined as the most potential cause.

Circa half of the patients (51.8%) declared that they received antiepileptic drugs (AEDs). In further details, 61.9% of them were on single-drug pharmacotherapy while 38% of the patients were on multiple-drug pharmacotherapy (11.9% of them received more than 3 AEDs) (Table 4).

**Table 4:** Epilepsy Pharmacotherapeutics in Iran.

**Received Antiepileptic medication**	(585) 51.8%	Single-drug pharmacotherapy	(362) 61.9%	Valproate sodium	13.6	22
				Phenytoin	11.7	19
				Carbamazepine	10.5	17
				Levetiracetam	9.9	16
				Lamotrigine	6.8	11
				Phenobarbital	4.3	7
				Others	4.9	8
				**Total**	**61.9%**	**100**
		Multiple-drug pharmacotherapy	(223) 38.0%	Phenytoin + Phenobarbital	7.6	20
				Valproate sodium + Carbamazepine	6.4	17
				Valproate sodium + Levetiracetam	6.4	17
				Others	14.8	39
				**Total**	**38.0%**	**100**
**Not Received Antiepileptic medication**			(545) 48.2%			

From the socio-economical aspect, our data disclosed that the out-of-pocket expenses of physician office visit/ ward round and medication were $76 per month (circa $0.71–$284.69 monthly).

## Discussion

This nationwide illustrative, population-based survey depicted that the prevalence of epilepsy in Iran in the present study population was 16.6 per 1000 people. The life time prevalence of epilepsy in this study was remarkably lower than other small conducted similar studies in Iran due to the large representative sample size that was used in the present study. Furthermore, our results concluded that the life time prevalence of epilepsy in Iran is superior to that compared with global life time prevalence of epilepsy in 2016^6^, probably due to the high prevalence of traumatic disasters especially road traffic injuries, occupational and the Iran-Iraq war injuries^7^. In addition, the life time prevalence of epilepsy in Iran was higher than developed countries including USA, Canada, Japan, France, Germany and Israel^6^. Moreover, in order to more accurate interpret our findings, the author have a tendency to compare our results with other nationwide studies (Table 5). Certainly, our neighbourhood countries particularly Turkey, Afghanistan and Iraq were exemplified to comparison due to our similarities in culture, healthcare system and socioeconomic states. However, lack of nationwide studies in neighbours and totally Middle East made it unachievable. Despite that, the increment in the rate of urbanization and westernization in Iran in the last 90 years, probably enhanced the comparison of Iran status with other western countries. As it was discussed previously, Beghi and colleagues estimated the global prevalence of epilepsy 0.6215% in 2016^6^. Their study mainly focused on idiopathic and conditions secondary to infectious diseases including meningitis, tetanus, malaria, cysticercosis^6^. Despite their finding, probably due to improvement in the control of infectious diseases in Iran^8^, our results did not conclude to a remarkable participation of these mechanisms in the life time prevalence of epilepsy in Iran. Nevertheless, the active prevalence of idiopathic epilepsy in Iran may be higher than the global, 2.7% against 0.038%, respectively^6^. As it was achieved by the present study, the prevalence of all active epilepsy (both idiopathic and secondary) was 9.5 persons per 1000 which is higher than the global prevalence of all active epilepsy in 2016 (6.215 persons per 1000)^6^. The prevalence of active epilepsy is highly variable between countries due to local distribution of risk factors. This point is exemplified by the paper of Beghi^9^, in which the prevalence of active epilepsy was compared between low/middle- and high-income countries. He concluded that the prevalence of active epilepsy in the low/middle-income countries was higher than high-income countries (8.75 vs. 5.18 persons per 1000, respectively). Therefore, socioeconomic risk factors may be the major determinant of the prevalence of active epilepsy. This point is clearly evident by Ngugi and colleagues’ paper^4^. They found not only the higher prevalence of active epilepsy in developing countries than developed countries but also higher prevalence in the rural areas of the developing countries as compared with the urban areas of the same countries.

**Table 5:** A comparison of epilepsy life time prevalence in nationwide scale in other countries/regions.

Study	Study year	Population size	Country/Region	Life-time prevalence (%)	Epilepsy types	Aetiologies
Beghi et al.^6^	2016	27,737,043	Global	0.6215	Idiopathic, Secondary	Meningitis, tetanus, malaria, cysticercosis, cystic echinococcosis, preterm birth complications, neonatal encephalopathy, neonatal sepsis, and neonatal haemolytic disease
Zack et al.^29^	2015	3,470,000	USA	1.2	Active	Not determined
Hamer et al.^30^	2009	634,566	Germany	0.91	Prevalence of patients receiving antiepileptic medication	Not determined
Serrano-Castro^31^	2012	1741	Spain	1.487	Partial seizure with/without secondary generalization, generalized tonic–clonic seizures, myoclonic seizures, idiopathic epilepsy, cryptogenic epilepsy	Not determined
Giussani et al.^32^	2011	912,458	Italy	0.79	Not determined	Not determined
Keränen et al.^33^	1989	2080	Eastern Finland	0.63	Active epilepsy, secondary to organic causes, generalized seizure, partial seizure, unclassified seizure	Not clarified
Joensen^34^	1986	43,609	Faroes, Denmark	0.78	Generalized epilepsy including primary (grand mal, petit mal, juvenile myoclonus) and secondary (west syndrome, Lennox-Gastaut syndrome), partial seizure	Not determined
Olafsson et al.^35^	1999	428	Rural Iceland	0.48	Partial seizure (simple partial, complex partial, partial secondarily generalized), primary generalized seizure (absence, myoclonic with or without other types, major motor seizure alone), other major motor seizures without aura, not classified	Idiopathic, remote symptomatic (cerebrovascular disease, MR/CP, infections, trauma), progressive symptomatic (primary and metastatic neoplasms), degenerative diseases (dementia)
Forsgren^36^	1992	713	Northern Sweden	0.55	Partial seizure (simple, complex, secondarily generalized), generalized seizure (tonic–clonic, myoclonic, absence, other), unclassifiable	Ischemic and haemorrhagic cerebrovascular disorders, trauma, tumour, infections, pre/perinatal asphyxia, prematurity, chromosomal aberration (Down syndrome, fragile X, (46XX, 13q +)), Rett syndrome, idiopathic
Onal et al.^37^	1999	2187	Rural areas of Istanbul, Turkey	0.8	Partial, generalized, unclassifiable	Not determined
Aziz et al.^38^	1997	24,130	Pakistan	0.99	Generalized tonic–clonic, simple partial, complex partial, generalized, absence, tonic and atonic, myoclonic	Idiopathic, past history of meningitis, encephalitis, neonatal jaundice, neonatal convulsions, hypertension, ischemic heart disease
Radhakrishnan et al.^39^	2000	238,102	Kerala, South India	0.49	Generalized, other	Not determined
Al Rajeh et al.^40^	2001	23,700	Saudi Arabia	0.654	Partial, generalized	Pre/perinatal encephalopathy, head injury, childhood neurological infection, stroke, febrile
Li et al.^41^	1983	63,195	China	0.44	Generalized nonconvulsive (akinetic, atonic), Generalized convulsive (grand mal), Partial epilepsy (with or without impairment consciousness, Multiple types	Brain injury, intracranial infection, and cerebrovascular disease
Guekht et al.^42^	2010	517,624	Russia	0.34	Generalized (Myoclonic,Atonic, Absence, Tonic, Tonic—clonic) Partial seizures (simple, Complex, Partial (simple and/ or complex) evolving to generalized)	Head injuries, cerebrovascular diseases, CNS infection, Pre/perinatal disorders, neurodegenerative disorders, tumours, unknown
Osuntokun et al.^43^	1982	18,954	Igbo-Ora, Nigeria	0.5	Generalized (Tonic–clonic, Petit mal,Grand mal, Partial (Simple, Complex),Unclassified	Not determined
Tekle-Haimanot et al.^44^	1986–1988	60,820	Meskan and Mareko, Ethiopia	0.52	Generalized tonic–clonic seizures, Partial, absence, unclassified	Not determined
Rwiza et al.^45^	1989	18,000	Ulanga, Tanzania	1.02	Partial (Simple, Complex, secondarily generalized), generalized (Absence, Tonic–clonic, Myoclonic, Tonic, Atonic), Unclassifiable	Idiopathic, Febrile convulsion, Unspecified encephalitis, Birth trauma, Cerebral malaria, Meningitis, Head trauma, Cerebrovascular disease, Suspected tumour
Birbeck et al.^46^	2000–2001	799		1.45	Not determined	Not determined

From the aspect of age and sex as risk factors that influence the life-time prevalence of epilepsy, we found that the life time prevalence of epilepsy was highest in the young who aged between 15 and 19 years old. Our finding is not exactly in harmony with previous studies. In other words, the life time prevalence of epilepsy was found to be lowest in the early life; nonetheless, we concluded that the life time prevalence of the epilepsy is lowest in the third decade of the life^1^. In addition to that, as it is depicted by our results, there was an increment trend toward the prevalence of epilepsy from the fourth decade to the sixth decade, then the life time prevalence of epilepsy reduced in the sixth decade of the life. Henceforth, the life time prevalence of epilepsy increased from the age 70 until the end of life. Despite our finding, similar studies in Europe and other industrialised countries revealed a decrement in the life time prevalence of epilepsy in 3^rd^ decade of life and a plateau state thereafter^1,10^.

Despite the above-mentioned findings in Europe, Weatherburn and colleagues disclosed an increment in the prevalence of epilepsy with age increment in Scotland in the population of older than 14 years old^11^. Nevertheless, we did not conclude to a remarkable difference among the children, younger, middle and elder adults, as it was reported a twofold higher life time prevalence of epilepsy in children and younger adults as compared with middle aged and elder adult in the middle east^12^. In this study, the mean age of onset of seizures was 19.1 years old which is in approximately in harmony with previous studies^13^.

This study is the first study in Iran that accurately assessed the life time prevalence in the age groups. Previous studies usually classified the prevalence of epilepsy by age as upper and lower 20 years old^14^. The main deficit of previous literature was that they did not provide a detailed life time prevalence estimation classified by age and sex, probably due to their limitation of sources and the size of their sample^15–18^. Hence, above-mentioned items were considered in the design of present study. In fact, there is a controversy that sex can influence the prevalence of epilepsy^19^. In the present study, it was revealed that epilepsy is more prevalent among male rather than female. This finding is in concert with previous literature^1,20^. Interestingly, the life time prevalence of epilepsy was remarkably higher in boys aged between 5 and 9 years than same-aged girls. In addition, men aged 15 to 19 year were more susceptible to epilepsy. However, women aged more than 80 years old showed a higher tendency toward epilepsy than other life period.

As it was not evident by previous literature, this study is distinguished from previous studies in providing of above-mentioned information. As compared with similar studies in the middle east, we also found that epilepsy life time prevalence is overall higher in male than female patients^12^. This finding may be attributable to the specific social and cultural atmosphere of Middle East, in which women encourage to conceal their diseases in order not to become isolated from the society and to improve their chance of marriage^19,20^.

In the present study also, we endeavoured to detect the prevalence of epilepsy as considered by types. Most of the Iranian patients in this study were diagnosed to suffer from focal epilepsy. This is interesting when generalized epilepsy is more prevalent in Middle East as it was evident by previous regional-wide studies^13^. The classification of epilepsy is highly dependent on the complex medical technology, and it may be the cause of differences among different studies.

This study is the first study in Iran that determined the exact pharmacotherapeutics received by patients. Most of the patients that were assessed in this study took a single drug monotherapy as it was revealed by previous studies in the middle east^13^. Previously, carbamazepine was determined to be the most common-prescribed AEDs, nonetheless, in this study we found that sodium valproate nowadays is the most administered drug. On the one hand, we determined that most of the Iranian patients suffered from focal epilepsy, but on the other, sodium valproate which is preferable drug for generalized seizure was the most common-used drug. As it was disclosed by previous studies, carbamazepine is highly effective for focal seizure therapy^21,22^.

Aetiologically, we found that trauma exemplified as head trauma and complications of neurological surgery was the most common underlying cause, however, previously, fever, tonic-colonic convulsions and epilepsy were found to be the predominant causes of epilepsy^5^. Our study was highly successful to determine an aetiology in circa half of the participants, while previous studies which assessed all age groups, concluded to a clear aetiology in 14% to 39% of cases^23^. To our knowledge, this study is the first study attempted to clarify the out-of-pocket expenses of patients with epilepsy in Iran. It is evident that epilepsy is more prevalent among low-income people^24^.

Our nationwide illustrative, population-based survey revealed that the life time prevalence of epilepsy in Iran was lower than other small conducted similar small studies in Iran. However, our results showed that the life time prevalence of epilepsy in Iran is higher than average worldwide prevalence of epilepsy. We hope that further investigations would be run to determine more precisely the effect of socio-economic status of patients with epilepsy on their prognosis and disease procedure in Iran.

## Methods and materials

### Study design

This study was designed as a population-based cross-sectional study. It was conducted from 2018 to 2021 in the both urban and rural regions of all provinces of Iran. The country's population according to last statistics provided by the United Nation data is 83,992,949 in 2020, of which 71.3% live in urban areas and 28.7% in rural areas. Iran is 1 648 195 km^2^ and consists of 31 provinces. There are more than 7 ethnic groups which approximately all are Caucasian-white.

### Sample size

In Previous study that has been conducted in Tehran, epilepsy life time prevalence estimated 10 per 1000 people^25^. Twenty-five thousand families were selected from 21,049,934 families in country through cluster sampling. In other words, there was 500 clusters of families consisted of 357 urban clusters and 143 rural clusters.

### Diagnostic criteria

According to the guidelines for epidemiologic studies on epilepsy^26^, epilepsy was defined as the condition characterized by recurrent (two or more) epileptic seizures, unprovoked by any immediate identified cause. Multiple seizures occurring in a 24-h period were considered a single event. Single epileptic seizures, and epileptic seizures with an obvious precipitant were excluded. Life time prevalence was a diagnosis of epilepsy (recurrent unprovoked seizures) at some point prior to the prevalence period or date. A prevalent case of active epilepsy was defined as a person with epilepsy who has had at least one seizure in the previous 5 years, regardless of antiepileptic drug treatment.

Seizures were classified in accordance with the international classification of epileptic seizures of ILAE^27^, based on the clinical history, age at onset, seizure patterns, evolution of the disease, clinical EEG and neuroradiologic examinations. A seizure was considered focal seizure on the basis of clinical evidence of focal onset, regardless of whether it was secondarily generalized. Remission referred to when epilepsy patients who have never been treated with any antiepileptic drugs were free of seizures for two years or more. Treatment gap was the number of people with active epilepsy not on adequate treatment, expressed as a percentage of total number with active epilepsy.

Related aetiology was accepted while there was strong association between cause and effect i.e., hospital recorded documents, localization (imaging and EEG) and/or time relevancy. Car accidents and war victims were well documented subjects in the head trauma category. If such a strong association was not found, the suspected risk factor was ignored. This strategy was followed by each etiological factor including trauma, stroke, infection, brain tumors and others. Self-report alone was not considered as sufficient criteria. Final decision for each case was made by an expert committee.

### Steps of the study

The present study used a two-phase door-to-door survey method. In phase I, a screening instrument for epilepsy was administered to subjects who agreed to participate; in phase II, the neurological evaluation of possible epilepsy was performed by expert neurologists among those subjects who screened positive for that condition in phase I.

### Phase I: screening

The field workers composed by local health staffs in the survey were given a standard training. The validated screening questionnaire by Placencia et al.^28^ was used to detect possible cases of epilepsy. The health workers who had been trained and could understand the screening questionnaires translated them into ethnic languages and dialects, if the people surveyed could not understand the screening questionnaires written in Persian. All staffs and interviews were trained and examined preceding the initiation of the study in order to ensure their qualifications required for the current study. The screening procedure was conducted during interview so that investigators had to verify that all subjects understood the questions asked in the questionnaire. Each interview was conducted by a native health staff due to the ethnic languages and dialects. Each adult resident in the house was interviewed. The head of the household, usually the husband, and his wife provided information about each child with age less than 14 years old. If necessary, the patients and their families were required to give a detailed demonstration of the seizure. In case of seizure history of family member(s), details were taken from the cases and also from a reliable eyewitness of the ictal event. The screening was completed when all subjects in a certain area were investigated.

### Phase II: Diagnosis and confirmation

Individuals whose responses to the questionnaire suggested they might have epilepsy were then scrutinized by a neurologist. Expert neurologists clinically examined these subjects at their residences, and reviewed relevant investigation records if available. The date of onset of seizure was ascertained as accurately as possible. On the basis of these observations, neurologists made the diagnosis of epilepsy and of other forms of seizure disorder and also identified and excluded the false positives. Most of patients with epilepsy were diagnosed through clinical evaluation and reviewing medical records. Experts from department of neurology together discussed the patients with unascertained diagnosis. As for the case of a disagreement among senior neurologists, the clinical, EEG and imaging data of the patients were discussed together to reach a consensus.

### Statistical analysis

Data were analysed through SPSS 18 (Chicago, IL). Chi-square and odd ratio were calculated in order to determine the correlation between epilepsy and demographic factors including sex and age.

### Ethics approval

The study was approved by the ethical committee of the Shahid Beheshti University of Medical Sciences (IRB code 1395.479). All participants, patients and interviewers gave written informed consent. Informed consent was given from the parents or legal guardians of participants who were under the codified age. All procedures performed in the present study were in accordance with the ethical standards of the institutional and national research committee of the Shahid Beheshti University of Medical Sciences and the 1964 Helsinki declaration and its further updates.
